# Fast and Easy Phage-Tagging and Live/Dead Analysis for the Rapid Monitoring of Bacteriophage Infection

**DOI:** 10.3389/fmicb.2020.602444

**Published:** 2020-12-18

**Authors:** Hui Zhi Low, Christina Böhnlein, Sabrina Sprotte, Natalia Wagner, Gregor Fiedler, Jan Kabisch, Charles M. A. P. Franz

**Affiliations:** Department of Microbiology and Biotechnology, Max Rubner-Institut, Federal Research Institute of Nutrition and Food, Kiel, Germany

**Keywords:** bacteriophage, flow cytometry, fluorescence, bacteriophage–host interactions, live–dead assay, *Pseudomonas fluorescens*, confocal laser scanning microscopy

## Abstract

Use of bacteriophages, which are viruses that kill bacteria, for biocontrol of pathogens and antimicrobial resistant bacteria has become increasingly important in recent years. As traditional culture-based methods are laborious and time-consuming, practicable use of bacteriophages will hinge on development of rapid and high throughput methods to analyze, characterize and screen large bacteriophage libraries. We thus established a novel method to fluorescently tag bacteriophages for virus screening and interaction studies, without the need for complicated and laborious purification procedures or genetic engineering of viruses to express fluorescent proteins. Bacteriophage PMBT14 was tagged using DNA dye Syto 13. Simply by using a membrane filter, tagged bacteriophages can be separated from non-sequestered excess dye rapidly, effortlessly, and cheaply. The procedure takes less than 30 min and makes use of simple laboratory consumables that are already commonly used for bacteriophage preparations. As proof of concept, we present here flow cytometric methods to analyze bacteriophage binding, infection and killing that are very accessible for high throughput analysis. We show that the resulting fluorescently tagged bacteriophage can be used to specifically stain its host bacterium *Pseudomonas fluorescens* DSM 50090. Individual fluorescent bacteriophages, their binding to and initial infection of bacteria could also be observed using confocal microscopy. The infection process was halted by the metabolic inhibitor sodium azide, suggesting a requirement of host metabolic processes for penetration by PMBT14. Flow cytometric live/dead assays was used as a complementary method to determine bacteriophage infection of its host. We made preliminary efforts to adapt the tagging method to two other bacteriophages and discuss potential pitfalls and solutions in the use of tagged phages. Fluorescent phage tagging has previously been demonstrated to facilitate analysis of bacteriophage–host interactions. The method adopted in this study makes it fast, easy as well as cost effective.

## Introduction

Bacteriophages, the mortal enemies of bacteria, have been increasingly recognized as a valuable tool in the biocontrol of both food spoilage as well as pathogenic bacteria. This is because they represent a natural and green technology compared to conventional heat and chemical decontamination processes for pathogen control (Moye et al., 2018). This is evidenced by the increasing number of commercial phage preparations that have been developed for combatting common food pathogens (Abuladze et al., 2008; Soni et al., 2010; Perera et al., 2015; Sukumaran et al., 2015; Grant et al., 2017).

Research on and the use of bacteriophages to combat problematic bacteria has been somewhat neglected since the discovery of antibiotics. The global annual World Health Organization estimates of deaths attributed to foodborne bacterial infections stands at 190,000, stemming from 350 million cases of foodborne bacterial infections occurring each year (Havelaar et al., 2015). Deaths attributed to diseases caused by antibiotic-resistant microorganisms stands at 700,000 per year. The alarming number of deaths caused by antimicrobial resistant bacteria could rise to 10 million per year by 2050 in the worst-case scenario, if no countermeasures are taken (Havelaar et al., 2015; IACG, 2019).

However, as bacteria become increasingly resistant to multiple antibiotics and we are left with little choices for their control, the use of bacteriophages as an option to control bacterial infection and contamination is increasingly becoming relevant (Gordillo Altamirano and Barr, 2019). Indeed, several studies have recently reported on the use of bacteriophages as a last resort to treat drug-resistant bacterial infections (Schooley et al., 2017; Dedrick et al., 2019). Furthermore, an increasing numbers of clinical trials have been initiated in recent years to study the topical and oral application of bacteriophages, with varying levels of success (Bruttin and Brüssow, 2005; Wright et al., 2009; Sarker et al., 2012, 2017; Gindin et al., 2019).

Because each bacteriophage can infect a narrow spectrum of bacteria, and bacteria can develop resistance to bacteriophages, it will be necessary to use a tailored cocktail of bacteriophages targeting different receptors on the bacteria to ensure maximum efficacy. Each potential bacterial pathogen will have to be tested against a library of bacteriophages to determine suitable candidates to be included in the treatment cocktail (Chan et al., 2013). This is currently very labor and time intensive, as decades old culture-based methodologies are still being widely used in which the host bacteria are required to first grow to confluence before growth inhibition, or lack thereof, can be observed. Advances in flow cytometry (FC) in terms of price, sensitivity, resolution of nanometer range particles and throughput have put the technique firmly on the landscape of microbiological research, even though it currently still remains a niche technique. Nevertheless, FC is already a very well-established technique used widely in biomedical research. The ability to analyze samples on the single cell level quickly and quantitatively, based on their light scatter and fluorescence characteristics, makes FC an extremely powerful technique to quantitatively measure bacteria counts and its viability status. DNA dyes, like Syto 9 and Syto 13, that acquire fluorescence only after binding to DNA, are commonly used in the staining and enumeration of bacteria (Stocks, 2004). As bacteriophages and viruses essentially consist of DNA or RNA encapsulated by proteins, they are also amenable to fluorescent labeling, thereby allowing them to be counted and tracked by FC. Going one step further, fluorescently tagged phages can be used to track and identify their host bacteria. Attempts to fluorescently tag bacteriophages for binding studies are not new (Goodridge et al., 1999; Mosier-Boss et al., 2003; Deng et al., 2012; Wu et al., 2016; Janczuk et al., 2017). However, the methods described are laborious, either requiring genetic engineering of the bacteriophages, or ultracentrifugation and/or dialysis/ultrafiltration to remove excess unbound dyes in the case of protein or nucleic acid staining approaches.

In this study a rapid and highly accessible method to fluorescently tag bacteriophages via their DNA with Syto 13 is presented. We also used a simple and fast live/dead stain using Syto 13 and propidium iodide (PI) to determine the host’s phage susceptibility. Using simple laboratory consumables, extraneous unbound dyes in tagged bacteriophage preparations that can unspecifically stain bacteria cells can be effortlessly depleted. As a proof-of-principle, a fluorescently tagged bacteriophage PMBT14 was used to visualize the infection of its host *P. fluorescens* DSM 50090, a common food spoilage microorganism, via flow cytometry (FC) and confocal laser scanning microscopy (CLSM). In addition, two other phages were tagged and we discuss possible problems and solutions that can occur when using tagged phages.

## Materials and Methods

### Microorganisms, Media and Buffers

The bacteria strains used in this study are listed in Table 2. Additional bacteria strains include *Pseudomonas aeruginosa* PAO 1 DSM 22644 and *Lactococcus lactis* F7/2 from our institute strain collection. Where indicated, bacteria with DSM numbers were obtained from the German Collection of Microorganisms and Cell Cultures. The virulent bacteriophage PMBT14 (Koberg et al., 2018) which belongs to the Siphoviridae was isolated from sewage collected from a wastewater treatment plant in the vicinity of Kiel using *P. fluorescens* (DSM 50090) as host bacterium. Phage incubation medium (PIM) consisted of 0.125× Ringer’s solution (Merck, Darmstadt, Germany), 20 mM glucose, 20 mM sodium pyruvate, 10% Brain Heart Infusion medium (BHI, Carl Roth, Karlsruhe, Germany) and 0.01% NaN_3_. For experiments with deviations in sodium azide concentrations, the sodium azide was either omitted (0%) or added at the concentration indicated. Phage acquisition medium (PAM) was comprised of 0.125× Ringer’s solution, 20 mM glucose, 20 mM sodium pyruvate and 1% BHI medium (Carl Roth). Additional virulent phage strains include JG004 (DSM 19871), belonging to the Myoviridae, that lyses *Pseudomonas aeruginosa* PAO 1 and P008, belonging to the Siphoviridae, that lyses *Lactococcus lactis* F7/2.

### Growth Conditions

All bacteria strains and phages were routinely cultured at room temperature (RT) in Caso broth and agar (Carl Roth, Karlsruhe, Germany), except for *L. lactis* F7/2, which was cultured in LM17 broth and agar at 30°C. Bacteria in 20% glycerol stocks were streaked onto agar plates and cultured for 1–3 days at room temperature (RT) before being stored at 4°C. Single colonies were inoculated from agar plates less than a week old into Caso broth and cultured overnight.

### Bacteriophage Stock

Bacteriophage PMBT14 and JG004 stock was generated using the confluent lysis and elution method (Sambrook and Russell, 2001) with some modifications. Briefly, bacteriophage lysates were diluted to the optimal concentration in 0.25× Ringer’s solution, of which 100 μL was added to 100 μL 40 mM CaCl_2_ and 300 μL overnight culture of host bacteria. After a 10 min incubation at RT, 3 mL of Caso soft-agar (0.75% agar) was added to the mixture and immediately poured onto a Caso agar plate. Bacteriophages from multiple plates were harvested after an overnight incubation at RT using SM buffer, filtered through 0.45 μm polyethersulfone (PES) syringe filter (Sarstedt, Nümbrecht, Germany), and concentrated by centrifugation at 50,000 × *g* for 2 h at 4°C. The bacteriophage pellet was then washed once with SM buffer supplemented with 0.04% gelatin (SMG) [+1% (v/v) Tween 20 (Carl Roth) in later preparations] and then twice with only SMG in a microcentrifuge at 18,000 × *g* for 2 h, before being resuspended in SMG. The bacteriophage stock was stored at 4°C or at −20°C by adding glycerol to a concentration of 10%. Phage P008 was prepared according to Müller-Merbach et al. (2005) and purified by cesium chloride ultracentrifugation (Sambrook and Russell, 2001).

### Syto 13 Tagging of Bacteriophage

One microliter of Syto 13 Green Fluorescent Nucleic Acid Stain stock solution (Life Technologies, Carlsbad, United States) was added to 20 μL of concentrated bacteriophage preparations, with titers typically in the range of 10^13^ PFU/mL. Bacteriophage was allowed to stain for 10 min at RT in the dark. The staining mix was further diluted 1:50–1:100 with SMG or SMG + 0.1%Tween (SMG-T) and allowed to equilibrate for 5 min before being slowly passed through a 0.45 μm PES filter membrane (VWR international, Catalog number 514-0074) to remove unbound Syto 13 dye. The absorption spectrum was measured using the Genesys 30 visible spectrophotometer (Thermo Scientific, Rockford, United States). Alternatively, when using more diluted bacteriophage stocks (10^11^ PFU/mL), 0.5–2 μL of Syto 13 stock solution was added to 1 mL bacteriophage in SMGT buffer and allowed to stain and equilibrate for 15 min with gentle end-over-end mixing before being filtered. Phages JG004 and P008 were also tagged this way. Because staining intensity decreased with storage, tagged bacteriophage preparations were used on the same day.

### Counting Beads

Accucheck and Countbright counting beads (Life Technologies) were used to determine flow rates for the FACSCalibur before each flow cytometric experiment in order to calculate accurate bacteria concentrations.

### Bacteria Staining With Syto 13 Tagged Bacteriophage PMBT14

Bacteriophage binding and infection was quantified via flow cytometry. Overnight cultures of bacteria were diluted 1:50 into PIM and processed within 15 min. Next, 40 μL of the 1:50 diluted bacteria were added to 10 μL of Syto 13 labeled bacteriophage (in SMG buffer) in a 96-well plate and incubated for 25 min at RT. In the meantime, live/dead analysis of the bacteria in PIM was performed by dilution of 4 μL into 246 μL live/dead staining solution (see below) and stained for 12 min at room temperature before acquisition on the FACSCalibur. At 25 min, the bacteria-bacteriophage mix was then further diluted 5 μL into 245 μL PAM, vortexed for 10 s and allowed to equilibrate at RT for 15 min before acquisition on the FACSCalibur flow cytometer (BD Biosciences, San Jose, United States). Multiple samples were processed in a time-delayed manner in order to ensure that every sample had the same incubation time until acquisition. No more than triplicates of six samples were processed at one time, with a 2-min delay between each sample.

### Live/Dead Lysis Assay

Live/dead analysis was performed for the quantification of total bacteria in bacteriophage tagging experiments, as well as to quantify phage lysis of host vs. non-host bacteria. Live/dead staining solution was prepared by diluting Syto 13 and 20 mM propidium iodide (PI) (Carl Roth) each 1:4000 in 0.25× Ringer’s solution. Live/dead analysis was performed by diluting bacteria suspension 1:50 into the live/dead staining solution and incubating for 10–12 min at RT before acquisition on the FACSCalibur. For quantification of phage lysis, overnight cultures of bacteria were diluted 1:50 into Caso broth and incubated with 10^8^ PFU/mL PMBT14 for 2 h before live/dead analysis. As negative control, no bacteriophage was added. After incubation, the physiological status of bacteria was analyzed by flow cytometric live/dead assay. A plate count of the bacteria was also performed using the spread plate method with 1:10 serial dilutions of the bacteria in 0.25× Ringer’s solution on Caso agar. For the quantification of phage lysis, a two-tailed Student’s *t*-test was performed for the plate count as well as each live/dead population to test for statistical significance between treatment with bacteriophage vs. no bacteriophage.

### Preliminary Bacteria Staining With Tagged Bacteriophages P008 and JG004

Staining of bacteria with tagged bacteriophage P008 and JG004 were performed with some adaptations, without live–dead staining. For *L. lactis* F7/2 staining, P008 Syto 13 (in SMG-T buffer) was incubated with bacteria for 5 min at RT before dilution in PAM and allowed to equilibrate for another 10 min before acquisition. Staining controls were done by using JG004 Syto 13 as background control and using *P. fluorescens* DSM50090 as non-host control to control for equivalent amounts of bacteriophage preparations. For *P. aeruginosa* PAO 1 DSM 22644, JG004 Syto 13 (in SMG-T buffer) was incubated with bacteria diluted in Caso broth for 60 min before dilution in PAM + 2.5 μM PI and acquired immediately with the FACSCalibur. Staining controls included the use of P008 Syto 13 as background control and *P. fluorescens* DSM50090 as non-host control.

### Fluorescence Emission Assay

In order to determine the amount of non-DNA bound Syto 13 in tagged PMBT14 bacteriophage preparations, we measured its fluorescence in the absence and presence of extraneous DNA. Free DNA was generated by lysing bacteriophage preparations (10^12^ PFU/mL in SMG-T buffer) at 95°C for 5 min. DNA concentration was measured using the Qubit DNA Assay (Thermo Fisher Scientific, Carlsbad, United States) to be 431 ng/mL. The free DNA solution was then diluted to 2.5, 12.5, and 25 ng/μL in SMG-T. 0 ng (buffer only), 5 ng, 25 ng, and 50 ng of DNA were then pipetted into a 96 well plate in 2 μL volumes. The tagged bacteriophage preparations were prepared either unfiltered, or filtered through a 0.45 μm syringe filter. The 0.45 μm filtered preparation was used without further treatment, additionally lysed (95°C for 5 min), or passed through a 0.2 μm syringe PES filter (Sarstedt). Syto 13 only solution with and without 0.45 μm filtration was used for comparison. For fluorescence measurement, 300 μL of the various bacteriophage/Syto 13 preparations were diluted in 1200 μL SMG-T and 100 μL was added to each well. Fluorescence in the wells was read using a TECAN Spark 10 M microplate reader (TECAN, Männedorf, Switzerland) at excitation and emission wavelengths of 450 nm and 510 nm, respectively.

### Standard Adsorption Assay

Adsorption of phage PMBT14 to *P. fluorescens* DSM50090 and *A. johnsonii* L2-215 was determined. Bacteria cell counts in overnight cultures were determined by live/dead staining and diluted into Caso broth to 2 × 10^8^ bacteria/mL. Bacteriophage PMBT14 was diluted into Caso broth +20 mM CaCl_2_ +5 mM MgSO_4_ to 2 × 10^7^/mL PFU/mL. 300 μL bacteriophage was added to 300 μL bacteria and allowed to adsorb for 25 min at RT. After adsorption, 10 μL of bacteria-phage suspension was diluted into 990 μL of 0.25× Ringer’s solution, vortexed for 10 s and centrifuged at 18,000 × *g* for 2 min to remove bacteria and bound bacteriophage. The unbound bacteriophage count was determined by soft agar overlay of serial dilutions of the supernatant with overnight culture of host bacteria. A one-way ANOVA statistical analysis was used to compare the means of the plaque forming units (PFU)/mL between no bacteria control and *P. fluorescens* DSM 50090 and *A. johnsonii* L2-215.

### Transmission Electron Microscopy (TEM)

Overnight culture of *P. fluorescens* DSM 50090 was diluted 1:50 in PIM. PMBT14 was diluted to 10^10^ PFU/mL in SMG buffer. 20 μL of diluted phage was mixed with 80 μL of diluted bacteria and allowed to adsorb for 25 min, with the last 20 min in the presence of thin carbon films which were floated from mica sheets into the bacteria/phage mixture. Transmission electron microscopy (TEM) and negative staining of phages bound to host cells was performed according to Ostergaard Breum et al. (2007) using a Tecnai 10 electron microscope (Philips, Eindhoven, Netherlands) with the acceleration voltage set at 80 KV.

### Confocal Laser Scanning Microscopy (CLSM)

Overnight cultures of *P. fluorescens* DSM 50090 and *P. fluorescens* L1-82 were pipetted into an 8-well μ-slide (Ibidi, Gräfelfing, Germany). As a non-*Pseudomonas* control, an overnight culture of *E. coli* was pipetted into a glass slide (Carl Roth) with a removable chamber (Ibidi). Bacteria were allowed to adsorb to the slide for 4–24 h at room temperature. Unadhered bacteria were then aspirated and the adhered bacteria washed once and then equilibrated with PIM + 3 μM *N*,*N*,*N*-trimethyl-4-(6-phenyl-1,3,5-hexatrien-1-yl) phenylammonium (*p*-toluenesulfonate (TMA-DPH) (Cayman, Ann Arbor, United States) for 10 min at room temperature. Staining solution was prepared by adding 60 μL Syto 13-tagged PMBT14 (SMG-T) to 240 μL PIM, with TMA-DPH added to end concentration of 3 μM. The equilibration solution was then replaced with the bacteriophage staining solution. Using a Leica SP8 DMI 6000 confocal microscope with a 63× objective and 5× zoom, a time course imaging of bacteriophage binding was performed in intervals up to 30 min on a constant field, and at 35 min a Z-stack was acquired on another field of view. Excitation wavelengths of 405 nm and 488 nm were used for Syto 13 and TMA-DPH, respectively.

### Efficiency of Plating Assays

Syto 13-tagged and untagged (without Syto 13) PMBT14 in SMGT buffer was processed as described above and were either unfiltered, or filtered through 0.45 and 0.2 μm PES syringe filters. Bacteriophage count was determined by soft agar overlay of serial dilutions of the preparation with overnight culture of host bacteria.

### Software

Flow cytometric data was analyzed using FCSExpress 6. Where indicated, statistical analyses were performed and graphs were generated using SigmaPlot. Confocal images were post-processed using ImageJ.

## Results

### Rapid Bacteriophage Tagging

**FIGURE 1:**
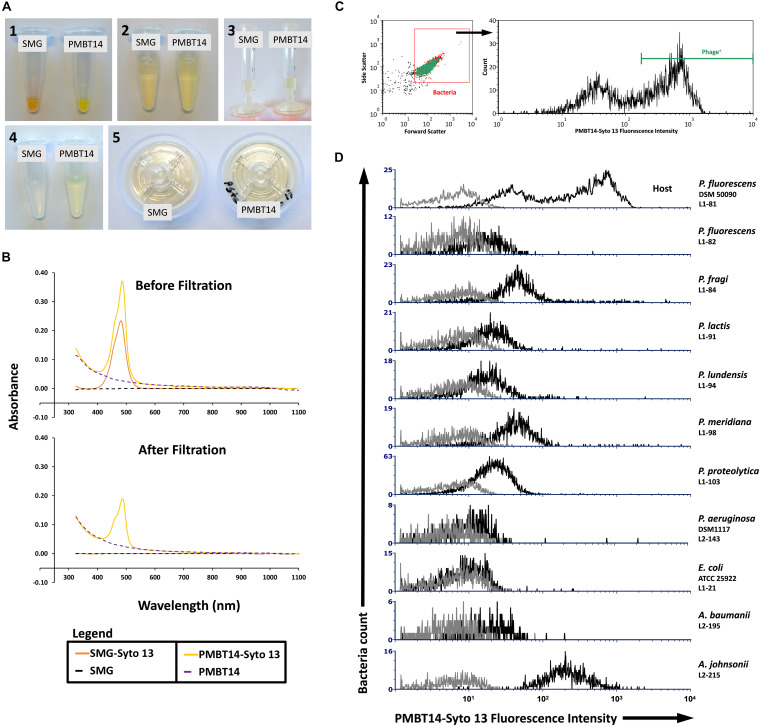
Syto 13 labeling of PMBT14 and its use in staining host bacteria. **(A)** Process of labeling bacteriophage using Syto 13 by **(1)** adding 1 μL Syto 13 to 20 μL 10^13^ PFU/mL bacteriophage (negative control SMG buffer only); **(2)** equilibrating the staining solution with 980 μL SMG buffer; **(3)** slowly passing the bacteriophage-Syto 13 mix through a 0.45 μm PES syringe filter; **(4)** filtrate; **(5)** retentate. **(B)** UV-Vis spectrophotometry of SMG buffer or PMBT14 treated with and without Syto 13, before and after filtration through the 0.45 μm filter. **(C)** Gating strategy for the flow cytometry analysis of bacteria staining with PMBT14-Syto13. Bacteria cells were first crudely gated based on their forward and sideward light scatter (left) and the infected “Phage^+^” cells were defined as cells that stained very positively with Syto 13 (right). **(D)** Histograms of PMBT14-Syto13 staining on host L1-81 *P. fluorescens* as well as a selection of other related and unrelated bacteria (black line). Unstained controls are in gray.

A rapid method was established to tag a bacteriophage using the fluorescent DNA binding dye Syto 13 in combination with a filtering step to remove unbound dye. In the absence of bacteriophage, Syto 13 in SMG buffer was fully retained on the 0.45 μm PES filter membrane, as evidenced by visual inspection (Figure 1A) and the complete removal of the 480 nm absorbance peak in the filtrate (Figure 1B, orange line). Bacteriophage tagged with Syto 13, however, was able to pass through the filter membrane, albeit with a lower 480 nm absorbance peak after filtration (Figure 1B, yellow line). The absorption spectra for SMG buffer and untagged PMBT14 are also shown (Figure 1B, black and blue dotted line, respectively). We did not observe significant changes in efficiency of plating of the bacteriophage in the presence of Syto 13 and also after filtration through 0.2 μm or 0.45 μm PES syringe filters (Table 1).

**TABLE 1:** Plating efficiency of PMBT14 on *P. fluorescens* DSM 50090 after Syto 13 staining and filtration.

**Staining**	**Filter**	**Titer (PFU/mL)**		**EOP**
		**Mean**	**SEM**	
No stain	No filter	3.81 × 10^11^	8.39 × 10^9^	1.00
	0.2 μm	3.96 × 10^11^	2.52 × 10^9^	1.04
	0.45 μm	4.37 × 10^11^	2.55 × 10^10^	1.15
Syto 13	No filter	4.26 × 10^11^	1.78 × 10^10^	1.12
	0.2 μm	4.61 × 10^11^	1.60 × 10^10^	1.21
	0.45 μm	3.44 × 10^11^	5.86 × 10^9^	0.90

*PFU, plaque forming unit; SEM, standard error of measurement; EOP, efficiency of plating; calculated by dividing mean titer of various treatment conditions against control that was unstained and unfiltered (first row).*

### Fluorescence Emission

Unbound Syto 13 has a low intrinsic fluorescence that makes it hard to detect in the presence of DNA-bound Syto 13 with a large fluorescence enhancement. Extraneous DNA was therefore added to the bacteriophage preparations to detect and semi-quantify the presence of unbound Syto 13 in bacteriophage preparations. This allows for a more precise determination of the efficacy of dye removal by the filter (Supplementary Figure S1). By looking at fluorescence increases (excitation 450 nm; emission 510 nm) from the addition of free DNA, it is possible to estimate the presence of free dye in the preparation. With increasing amounts of DNA, Syto 13 in SMG-T buffer became increasingly fluorescent. 100 μL of the 1:5 diluted Syto 13 in SMG-T buffer solution gave a mean fluorescence increase of 8827 in the presence of 50 ng DNA, when compared to sample without DNA. Henceforth, this value was termed as ΔEm_510_ (fluorescence-increase from addition of 50 ng DNA) (Supplementary Figure S1). After filtration through 0.45 μm filter, any intrinsic fluorescence as well as fluorescence increase was almost completely eliminated. PMBT14 incubated with Syto 13 (before filtration) had a high intrinsic fluorescence due to the bacteriophage particles and a ΔEm_510_ of 10540. After 0.45 μm filtration however, the intrinsic fluorescence decreased and ΔEm_510_ was lowered to 3813. Further filtration using a 0.2 μm filter decreased the intrinsic fluorescence as well as the ΔEm_510_ even further to 1611. Heat lysis of the 0.45 μm filtered PMBT14-Syto 13 led to a massive increase in intrinsic fluorescence, but the ΔEm_510_ (-374) did not increase with additional DNA.

### Quantification of Bacteria Staining With Tagged PMBT14 by Flow Cytometry

Bacteria cells were loosely gated by their forward and sideward scatter, and then analyzed for their Syto 13 fluorescence as indication for their binding to and infection by Syto 13-labeled bacteriophage PMBT14 (PMBT14-Syto 13). Only the strongly Syto 13 fluorescent bacteria (fluorescence intensity > 350) were considered as having bound to and been infected by PMBT14-Syto 13, and are thus labeled as phage positive (Phage^+^) (Figure 1C). In host *P. fluorescens* DSM 50090, 64% of the bacterial population was positively stained (Figure 1D and Table 2). Using PMBT14 tagged with Syto 13 in SMG, an optimized binding protocol and identical gating strategy between samples, we observed very strong fluorescence in the host *P. fluorescens* DSM 50090. Very little unspecific staining occurred in most other negative control bacteria, as demonstrated by the very low counts within the Phage^+^ gate, including *Listeria innocua* 6a, *Escherichia coli*, as well as 27 *Pseudomonas* spp. and 7 *Acinetobacter* spp. strains (Figure 1D and Table 2). Unspecific staining was observed only for both of the tested *Acinetobacter johnsonii* strains L2-208 and L2-215. Although there was some negligible background noise in the no bacteria control, we did not see any Phage^+^ staining.

**TABLE 2:** PMBT14-Syto 13 binding to the bacteria strains tested in this study.

**Internal number**	**Bacteria strain**	**Source**	**Total bacteria conc.**	**Dead bacteria conc.**	**Dead bacteria (%)**	**Phage^+^ conc.**	**Phage^+^ (%)**
							
			**(×10^7^/mL)**	**(×10^7^/mL)**		**(×10^7^/mL)**	
							
			**2 replicates**	**2 replicates**		**3 replicates**	
							
			**Mean** ± **SEM**	**Mean** ± **SEM**		**Mean** ± **SEM**	
n.a.	No bacteria control	n.a.	0.01 ± 0.00	0.00 ± 0.00	36.8	0.00 ± 0.00	0.0
L1-81	*Pseudomonas fluorescens*	Freshwater prefilter tanks (DSM 50090)	5.17 ± 0.35	0.10 ± 0.01	1.9	3.31 ± 0.18	64.1
L1-82	*Pseudomonas fluorescens*	Raw milk	3.39 ± 0.06	0.08 ± 0.00	2.2	0.00 ± 0.00	0.0
L1-83	*Pseudomonas fluorescens*	Raw milk	3.81 ± 0.05	0.01 ± 0.00	0.4	0.01 ± 0.00	0.2
L1-84	*Pseudomonas fragi*	Raw milk (DSM 3456)	2.68 ± 0.12	0.04 ± 0.00	1.5	0.11 ± 0.00	3.9
L1-85	*Pseudomonas fragi*	Raw milk	6.13 ± 0.34	0.01 ± 0.00	0.1	0.01 ± 0.00	0.2
L1-86	*Pseudomonas fragi*	Raw milk	0.86 ± 0.03	0.02 ± 0.01	1.8	0.01 ± 0.00	0.8
L1-87	*Pseudomonas fragi*	Raw milk	0.45 ± 0.03	0.01 ± 0.01	2.7	0.01 ± 0.00	2.8
L1-88	*Pseudomonas gessardii*	Mineral water (DSM 17152)	3.00 ± 0.01	0.02 ± 0.00	0.6	0.04 ± 0.00	1.2
L1-89	*Pseudomonas gessardii*	Raw milk	7.67 ± 0.32	0.04 ± 0.01	0.5	0.02 ± 0.00	0.2
L1-90	*Pseudomonas gessardii*	Raw milk	1.94 ± 0.02	0.01 ± 0.00	0.7	0.03 ± 0.00	1.5
L1-91	*Pseudomonas lactis*	Raw milk	2.65 ± 0.13	0.03 ± 0.00	1.3	0.01 ± 0.00	0.5
L1-92	*Pseudomonas lactis*	Raw milk	5.55 ± 0.10	0.08 ± 0.00	1.4	0.04 ± 0.00	0.7
L1-93	*Pseudomonas lactis*	Raw milk (DSM 29167)	1.70 ± 0.01	0.03 ± 0.00	2.0	0.02 ± 0.00	0.9
L1-94	*Pseudomonas lundensis*	Prepacked beef (DSM 6252)	2.68 ± 0.09	0.03 ± 0.00	1.0	0.02 ± 0.00	0.6
L1-95	*Pseudomonas lundensis*	Raw milk	4.60 ± 0.02	0.03 ± 0.00	0.6	0.01 ± 0.00	0.1
L1-96	*Pseudomonas lundensis*	Raw milk	1.29 ± 0.07	0.04 ± 0.00	2.9	0.01 ± 0.00	0.4
L1-97	*Pseudomonas lundensis*	Raw milk	0.55 ± 0.02	0.00 ± 0.00	0.7	0.00 ± 0.00	0.7
L1-98	*Pseudomonas meridiana*	Raw milk	1.56 ± 0.03	0.03 ± 0.00	1.8	0.03 ± 0.00	2.1
L1-99	*Pseudomonas meridiana*	Raw milk	7.07 ± 0.51	0.27 ± 0.01	3.8	0.12 ± 0.00	1.7
L1-100	*Pseudomonas protegens*	Raw milk	5.03 ± 0.86	0.12 ± 0.01	2.3	0.06 ± 0.01	1.2
L1-101	*Pseudomonas protegens*	Raw milk	5.22 ± 0.38	0.23 ± 0.02	4.3	0.04 ± 0.00	0.7
L1-102	*Pseudomonas protegens*	Raw milk	2.86 ± 0.08	0.05 ± 0.01	1.6	0.11 ± 0.01	3.8
L1-103	*Pseudomonas proteolytica*	Cyanobacterial mat samples (DSM 15321)	6.48 ± 0.16	0.02 ± 0.00	0.3	0.02 ± 0.00	0.3
L1-104	*Pseudomonas proteolytica*	Raw milk	8.86 ± 0.60	0.02 ± 0.00	0.2	0.02 ± 0.00	0.2
L1-105	*Pseudomonas proteolytica*	Raw milk	1.95 ± 0.06	0.01 ± 0.00	0.4	0.01 ± 0.00	0.4
L1-106	*Pseudomonas proteolytica*	Raw milk	11.50 ± 0.26	0.05 ± 0.00	0.4	0.02 ± 0.00	0.2
L1-107	*Pseudomonas proteolytica*	Raw milk	9.78 ± 0.08	0.02 ± 0.00	0.2	0.02 ± 0.00	0.2
L2-195	*Acinetobacter baumannii*	Raw milk	0.91 ± 0.05	0.00 ± 0.00	0.1	0.00 ± 0.00	0.0
L2-196	*Acinetobacter calcoaceticus*	Raw milk	0.79 ± 0.01	0.00 ± 0.00	0.3	0.00 ± 0.00	0.2
L2-197	*Acinetobacter pittii*	Raw milk	1.40 ± 0.26	0.01 ± 0.00	0.6	0.00 ± 0.00	0.0
L2-198	*Acinetobacter baumannii*	Raw milk	1.00 ± 0.03	0.00 ± 0.00	0.3	0.01 ± 0.00	0.5
L2-199	*Acinetobacter baumannii*	Raw milk	0.98 ± 0.02	0.01 ± 0.00	0.6	0.00 ± 0.00	0.4
L2-203	*Acinetobacter hemolyticus*	Raw milk	1.53 ± 0.06	0.00 ± 0.00	0.2	0.00 ± 0.00	0.0
L2-207	*Acinetobacter junii*	Raw milk	0.47 ± 0.00	0.00 ± 0.00	0.5	0.01 ± 0.00	2.1
L2-208	*Acinetobacter johnsonii*	Raw milk	1.03 ± 0.01	0.01 ± 0.00	0.6	0.07 ± 0.01	6.6
L2-215	*Acinetobacter johnsonii*	Raw milk	1.57 ± 0.00	0.00 ± 0.00	0.2	1.04 ± 0.02	66.2
L2-143	*Pseudomonas aeruginosa*	DSMZ (DSM 1117)	1.13 ± 0.04	0.01 ± 0.00	0.9	0.00 ± 0.00	0.1
L2-236	Enteroaggregative *Escherichia coli*	Raw milk	3.38 ± 0.20	0.01 ± 0.00	0.4	0.01 ± 0.00	0.2
L1-21	*Escherichia coli*	ATCC (ATCC 25922)	2.84 ± 0.05	0.01 ± 0.00	0.3	0.00 ± 0.00	0.1
L1-63	*Listeria innocua 6a*	DSMZ (DSM 20649)	4.78 ± 0.10	0.01 ± 0.01	0.3	0.00 ± 0.00	0.0

*Total and dead bacteria concentration were obtained from live/dead staining. Phage^+^ concentration is the concentration of bacteria gated as Phage^+^ (Figure 1C) and the Phage^+^ (%) is calculated from Phage^+^/total bacteria concentration. n.a., not applicable.*

### Visualizing Bacteriophage Infection

**FIGURE 2:**
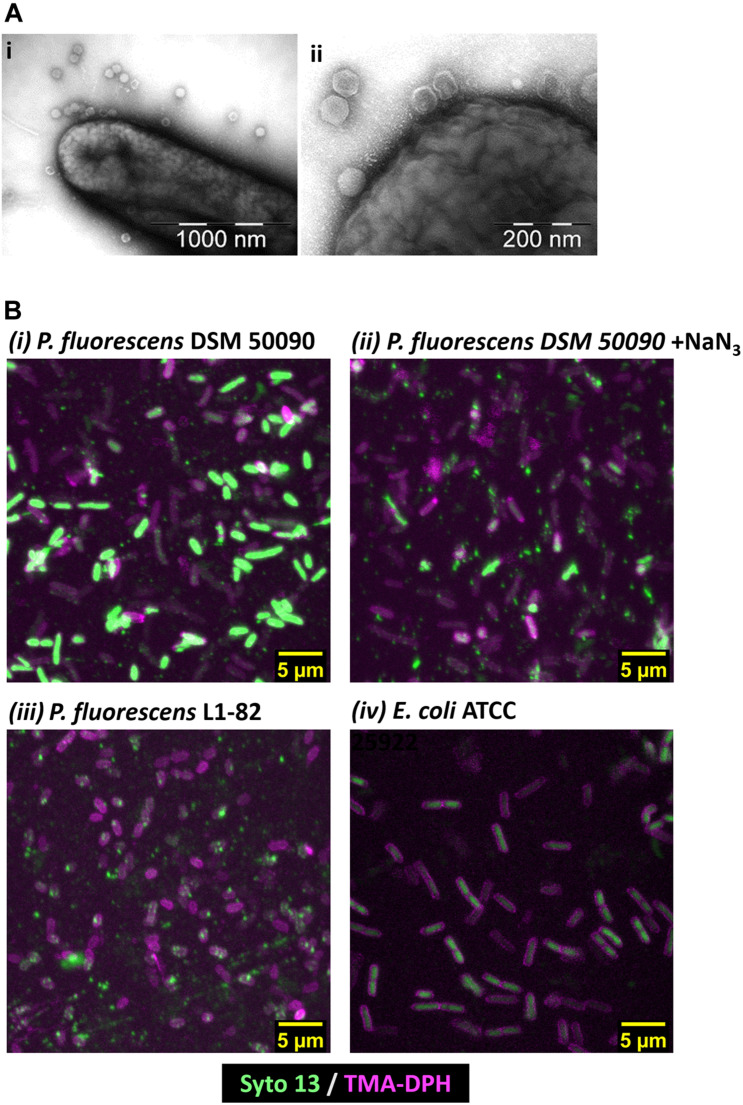
Microscopy of PMBT14 binding to and infection of its host. **(A)** TEM microscopy of PMBT14 binding to the host. **(B)** Maximum intensity projection of Z-stack acquired via CLSM after 35 min incubation of PMBT14-Syto 13 with bacteria. PMBT14-Syto 13 binding to and infection of its host *P. fluorescens* DSM 50090 (UL). Binding but attenuated infection in the presence of 1% NaN3 (UR), and no infection in *P. fluorescence* L1-82 (LL). No binding or infection with PMBT14-S13 occurred in *E. coli* L1-21(LR). 3D projections of the Z-stacks are can be seen under Supplementary Videos.

Using TEM, we observed direct binding of multiple PMBT14 phages to its host *P. fluorescens* DSM 50090 (Figure 2A). Using PMBT14-Syto 13, we also visualized the infection of its host bacteria via CLSM. Bacterial cells were adhered onto a coverslip and stained with TMA-DPH membrane dye, outlining the individual bacteria cells in magenta (Figure 2B). Supplementary Videos S1–S4 allow the 3D visualization of phage-binding to bacteria cells. Strong Syto 13 staining of individual bacteriophage particles was observed as small green fluorescent dots that associated with the bacteria cells. However, only in the sample with *P. fluorescens* DSM 50090 was the green fluorescence of PMBT14-Syto 13 able to penetrate the cell and transfer to a substantial portion of the bacteria (Figure 2Bi, Supplementary Figure S2, and Supplementary Video S1). We henceforth defined this population of bacteria as “infected.” This corresponded to a substantial increase in fluorescence intensity quantified via flow cytometry, with 64% of the bacteria population infected with PMBT14-Syto 13 (Figure 1D and Table 2). PMBT14-Syto 13 was also observed to bind to another non-host *P. fluorescens* L1-82 strain under CLSM, whereby no transfer of green fluorescence occurred (Figure 2Biii, Supplementary Figure S2, and Supplementary Video S3). However, this binding was not captured via flow cytometry (Figure 1D). Increasing concentrations of sodium azide to 0.1–1% led to inhibition of infection in *P. fluorescens* strain L1-81 (Figures 1D, 2Bii, Supplementary Figure S2, and Supplementary Video S2). As a negative control for binding and staining, the unrelated Gram-negative bacterium *Escherichia coli* L1-21 ATCC 25922 neither bound to any green fluorescent bacteriophage particles nor acquired significant Syto 13 fluorescence using FC or CLSM (Figures 1D, 2Biv, Supplementary Figure S2, and Supplementary Video S4).

### Characterization of Unspecific Staining of *A. johnsonii* L2-215

**FIGURE 3:**
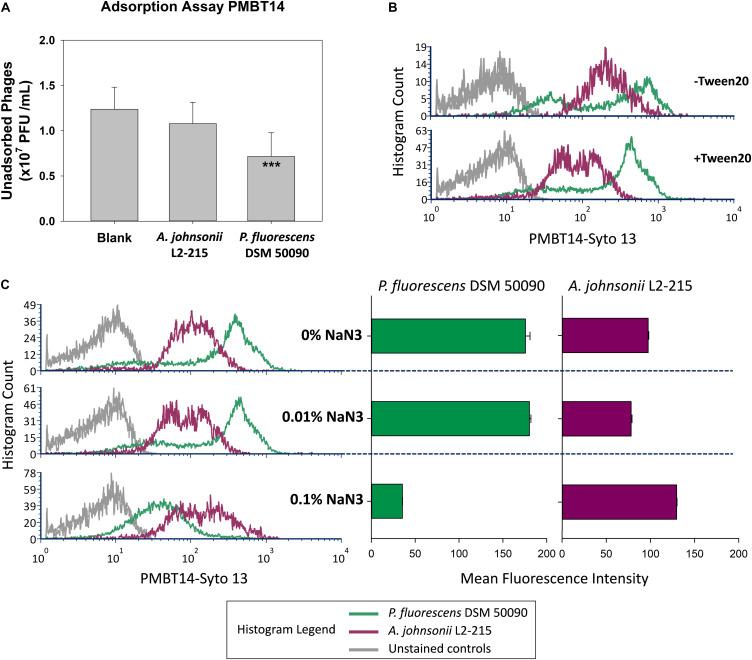
Characterization of unspecific staining in *A. johnsonii*. **(A)** Standard adsorption assay of PMBT14 using *A. johnsonii* L2-215 and host *P. fluorescens* DSM 50090. Bar graphs depict the amount of unadsorbed bacteriophage (*n* = 12 from four experiments). One-way ANOVA was used to compare the means between the groups (2 degrees of freedom; *F*-value 13.968; *p < 0.05; **p < 0.01; ***p < 0.001). **(B)** Staining intensity of Tween 20 treated (+Tween 20) PMBT14-Syto 13 versus original untreated PMBT14-Syto 13 (–Tween 20). **(C)** Histograms (left) depicting the effect of increasing concentrations of sodium azide on the fluorescence of *P. fluorescens* DSM 50090 and *A. johnsonii* L2-215 after incubation with PMBT14-Syto 13. Bar graphs (right) depict the corresponding mean fluorescence intensity with SEM (*n* = 3).

The unspecific staining of *A. johnsonii* L2-215 was investigated more closely. Classical adsorption assays revealed that while the host bacteria were able to bind to and thereby significantly deplete PMBT14 phages, *A. johnsonii* L2-215 was not (Figure 3A). When the bacteriophage preparation was washed with 1% Tween in SMG and 0.1% Tween was included in the bacteriophage tagging solution, a substantial, but incomplete, reduction of unspecific staining of *A. johnsonii* L2-215 could be observed, as well as a better separation in the fluorescence signal intensity between *A. johnsonii* L2-215 and host *P. fluorescens* DSM 50090 (Figure 3B). Furthermore, with increasing concentrations of sodium azide in the incubation solution, staining of host *P. fluorescens* DSM 50090 was abolished, whereas that of *A. johnsonii* L2-215 was not (Figure 3C).

### Lysis Assay

**FIGURE 4:**
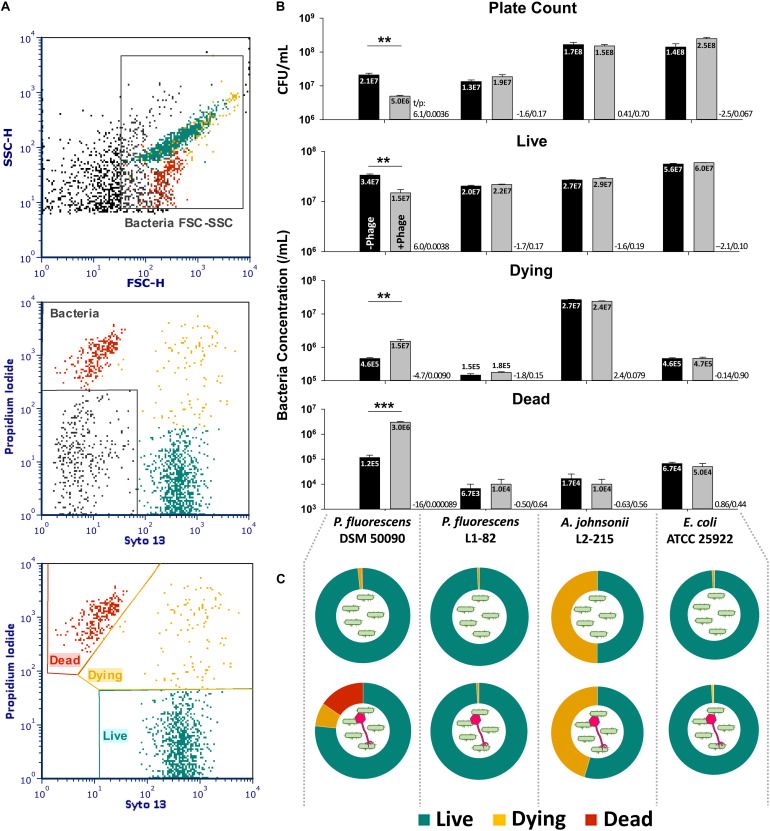
Characterization of phage lysis using live/dead cell viability assay. **(A)** Gating strategy for the live/dead assay. Bacteria cells were first crudely gated based on their forward and sideward light scatter (top), and further refined from debris based on their staining for Syto 13 and/or PI (middle). The bacteria population was then classified into three physiological statuses based on their staining characteristics (bottom). **(B)** Concentration of bacteria cells of various physiological statuses with SEM (*n* = 3 from one experiment, representative data from two independent experiments) after a 2-h incubation with 10^8^ PFU/mL PMBT14 (gray bars) or no phage (black bars). Mean of each population is given in the bars in scientific notation. Two-tailed Student’s *t*-test was performed for each population to test for statistical significance between treatment with bacteriophage versus no bacteriophage. *t*-Value (*t*) and *p*-value (*p*) is given next to each pair analyzed (*p < 0.05; **p < 0.01; ***p < 0.001). **(C)** Pie chart representations of bacteria physiological status after 2 h incubation without phage (top row) and with phage PMBT14 (bottom row).

A lysis assay using live/dead staining to confirm bacteriophage activity on different bacteria was also performed. The stained bacteria could be separated into “live” (Syto 13^hi^, PI^lo^), “dying” (Syto 13^hi^, PI^hi^) and “dead” (Syto 13^lo^, PI^hi^) populations (Figure 4A). A decrease in live cell count and an increase in the dead and dying cell count in comparison to no-phage control would indicate that lysis or killing has occurred. Optimization of lysis conditions determined that 10^8^ plaque-forming units (PFU)/mL of PMBT14 were required to obtain a good lysis response after 2 h on approximately 10^7^/mL (MOI ∼10) of the host bacteria using flow cytometry. In the host *P. fluorescens* DSM 50090, 2-h incubation with PMBT14 led to a significant increase in dead and dying cells, with a concomitant decrease in live cells in comparison to negative controls without bacteriophage (Figures 4B,C). The change in live cell count was comparable to the plate count obtaining using the spread plate method. In non-host *P. fluorescens* L1-82, *A. johnsonii* L2-215 and *E. coli*, no such changes in live counts could be observed.

### Bacteria Staining With Tagged P008 and JG004

We adapted the tagging and staining method to bacteriophages P008 and JG004. *P. fluorescens* DSM 50090 was used as a non-host control to validate that similar amounts of tagged P008 and JG004 bacteriophage preparations were being used (Supplementary Figures S3, S4). For *P. aeruginosa* PAO1, the intrinsic dead cell population was stained with PI and excluded from analysis. Through that, we were able to observe significantly higher staining of *P. aeruginosa* PAO 1 by its cognate phage JG004-Syto 13 as compared to background control P008-Syto 13 (Supplementary Figure S3). For *L. lactis* F7/2, the high background fluorescence indicated by background control JG004-Syto 13 was nevertheless superseded by staining with its cognate phage P008-Syto 13 (Supplementary Figure S4).

## Discussion

In this study, a simple, effortless and non-destructive method to fluorescently label bacteriophages and to deplete excess unbound dye, using standard laboratory materials, was established and described in detail. Using a tagged bacteriophage, it was possible to easily and clearly track the infection of its host bacterium, using both flow cytometry and confocal fluorescence microscopy. In the process, we also used a quick and simple live/dead assay to determine phage susceptibility.

In the course of establishing flow cytometry protocols to stain and enumerate bacteria, the phenomenon that the Syto dyes, Syto 9 and Syto 13 were not amenable to membrane filtration and were retained on the filter, was observed. It has recently been described that the hydrophobic fluorescent DNA dye SYBR Green I was able to form auto-fluorescent colloidal particles in aqueous solutions, and that these were unable to pass through 0.22 μm sterilizing filter membranes (Dlusskaya et al., 2019). The chemical structure of the Syto dyes are proprietary and therefore not public knowledge. Similar to SYBR Green I, they are cyanine dyes that are able to penetrate intact cell membranes, suggesting that they must also possess a hydrophobic nature. We suspect that, similar to SYBR Green I, Syto 13 forms colloids when diluted in buffer, which is then retained on the filter membrane. When Syto 13 binds to bacteriophage DNA and is sequestered inside of a bacteriophage, it loses its colloidal nature and this enables the dye to pass through the filter. Using this phenomenon, we devised the bacteriophage staining method using 0.45 μm membrane filters to remove extraneous unbound Syto 13 from Syto 13 tagged bacteriophage, requiring only standard laboratory consumables and taking only 20–30 min, with 5 min of hands-on time.

The 0.45 μm PES syringe filter was clearly able to effectively remove unbound Syto 13 in buffer as evidenced by the disappearance of the absorption peak and fluorescence emission in presence of DNA (Figure 1B and Supplementary Figure S1). However, Syto 13 removal in bacteriophage preparations was less clear cut. The PMBT14 and JG004 bacteriophage preparations in this study were only purified by centrifugation and there could have been substantial contaminants present, including extracellular DNA and lipid containing fractions, like endotoxins and vesicles, that could also have unspecifically sequestered Syto 13 and passed through the filter. It is also possible that unbound Syto 13 could be sequestered in the bacteriophage heads in spaces between the DNA. The presence of contaminating Syto 13 in the bacteriophage preparations that was not bound to bacteriophage DNA could, therefore, not be excluded. Indeed, addition of 50 ng DNA led to a further increase in fluorescence of 100 μL of the tagged bacteriophage preparations. This ΔEm_510_ was nevertheless much smaller than the increase before filtration was carried out, indicating removal of free dye. Filtration of the tagged bacteriophage preparation with a 0.2 μm filter decreased the ΔEm_510_ even further, but had no effect on the efficiency of plating. This indicates that a smaller pore size can deplete extraneous Syto 13 more efficiently. Heating the tagged bacteriophage preparations to 95°C destroyed the bacteriophages as well as any contaminating vesicles able to sequester unbound Syto 13, resulting in a massive increase in fluorescence as it comes in contact with freed phage DNA. Nevertheless, even though the Syto 13 removal appears incomplete in tagged bacteriophage preparations, this was enough to deny fluorescence to 27 *Pseudomonas* spp., 10 *Acinetobacter* spp. strains, *E. coli* and *L. innocua* (Figure 1D and Table 2). This shows that the Syto 13 in vesicles or capsid heads remain mostly sequestered away after filtration, thereby preventing access to bacteria cells.

Using the tagged bacteriophage PMBT14, we observed a penetration of green fluorescence into its host *P. fluorescens* L1-81 via CLSM. This is most likely due to the initial process of bacteriophage infection, whereby the bacteriophage penetrated the host membrane. An explanation for that is that the green fluorescent DNA in bacteriophages might have been injected from the bacteriophage into the host. However, due to the presence of unbound Syto 13, it is possible that unbound Syto 13 also entered the bacteria through the weakness in the membrane on account of bacteriophage penetration. Interestingly, attachment of PMBT14 to another non-host *P. fluorescens* L1-82 strain, whereby no penetration occurred, could also be observed using CLSM (Figure 3Biii). This is likely a direct observation of reversible phage binding which was not captured via flow cytometry due to a 10 s vortex step that disrupts reversibly bound bacteriophage (Figure 1D and Table 2). Increasing concentrations of the metabolic poison sodium azide led to a significant decline in infection of *P. fluorescens* DSM50090 with PMBT14-Syto13 under flow cytometry and CLSM (Figures 1D, 3Bii), indicating that infection by the bacteriophage requires host metabolic processes.

Due to presence of residual Syto 13, some level of background was observed in the Z-stack obtained from incubation of *E. coli* with PMBT14-Syto 13. We did not observe any such background during the time course as well as during flow cytometry (Supplementary Figure S2, Figure 1, and Table 2). TMA-DPH tends to induce cell death among the bacteria when used in excessive amounts and during prolonged UV irradiation (own observation). This might explain a higher background during the acquisition of the Z-stack, whereby multiple images were scanned from the same field of view. In the future, it would be worth exploring other less toxic counterstains. In addition, because *E. coli* did not adhere to plastic, glass coverslips had to be used instead of plastic coverslips, which might have had slightly different transmission properties. However, we simply viewed this as background because we did not observe an increase in fluorescence under flow cytometry and that the infected bacteria in *P. fluorescens* DSM 50090 was much more fluorescent.

All our bacterial cultures were freshly cultured from a single bacteria colony. However, even in such an apparently genetically homologous population of bacteria, it is clear that there exist heterogeneous subpopulations with differing phenotypic binding and permissiveness to infection, i.e., phage-sensitivity. Although the majority of *P. fluorescens* DSM50090 cells were infected by fluorescent PMBT14, there persistently was a sizeable population that was not infected, indicated in this study using FC by the negative peak with lower fluorescent intensity (Figure 1D). This is also reflected in the CLSM observation of PMBT14 binding to its host, whereby a substantial proportion of the bacteria captured under the microscope remained uninfected (Figure 3B). It is evident in this case that not every host bacterium expresses the bacteriophage receptor or is permissive to infection. Permissiveness of a host to infection under different conditions is a fundamental question that has been very difficult to answer using classical experimental approaches (Dang and Sullivan, 2014). Nevertheless, it is crucial for evaluating the effectiveness of bacteriophages in the context of its environment, and eventually, in its use in therapy and biocontrol. These are fundamental questions that may be better resolved when binding and infection of the host by its bacteriophage (or lack thereof) can be directly and readily observed and quantified.

We made preliminary attempts to adapt the tagging and staining protocol to other virulent phages, including JG004 (host: *P. aeruginosa* PAO1) and P008 (host: *L. lactis* F7/2). In order to make the staining more robust against physiological differences between different bacteria that can lead to differing unspecific or background fluorescence, unrelated bacteriophages that should not bind to the host bacteria were used as background control. This is analogous to using isotype controls when performing immunological assays. In using unrelated bacteriophages, the host bacteria is its own control for unspecific staining. In this case, a non-host control is used to check for equivalent amounts of cognate and unrelated bacteriophages. For staining of *P. aeruginosa* PAO 1, phage P008 was used as background control. By using such controls, we realized that *P. aeruginosa* has a steady population of dead cells that is highly prone to unspecific staining with phage P008 that can hinder specific analysis of phage infection (own observation). We therefore added PI to the PAM to stain for and gate out the dead cells. With this method, unspecific staining due to the substantial dead cell population was mitigated by gating out the dead cell population. *P. aeruginosa* PAO1 staining by JG004-Syto 13 could then be distinguished from non-specific staining by bacteriophage P008-Syto 13 (Supplementary Figure S3).

*Acinetobacter johnsonii* itself stains very strongly for Syto 13 (data not shown). Although the non-specifically sequestered Syto 13 did not seem to cause a problem in most negative control bacteria, it appears to be problematic for *A. johnsonii* L2-215 which also appeared positively stained (Figure 1D and Table 2). PMBT14 did not appear to adsorb to or lyse *A. johnsonii* L2-215 (Figures 3A, 4). Unlike in *P. fluorescens* DSM50090, unspecific fluorescence in *A. johnsonii* L2-215 remained high with increasing sodium azide concentrations. We can only conclude that it is the presence of residual Syto 13 that is responsible for this unspecific staining. Washing the bacteriophage preparations with 1% Tween 20 and using 0.1% Tween in bacteriophage labeling led to a substantial, but still incomplete reduction of fluorescence signal in *A. johnsonii* L2-215, but allowed a better fluorescence separation from the host *P. fluorescens* DSM50090. This points to lipid containing fractions to be, at least partially, possible causes for residual Syto 13 that can cause unspecific staining. Extracellular DNA was excluded as a major contaminant as DNAse I treatment of bacteriophages did not abolish this unspecific staining in *A. johnsonii* L2-215 (data not shown).

For staining of *L. lactis* F7/2, phage JG004 was used as background control. When attempting to use tagged phage P008 to stain *L. lactis* F7/2, we also observed an increase in background staining in *L. lactis* F7/2 when using unrelated phage JG004 (Supplementary Figure S4). This represents a major weakness of using bacteriophages tagged with DNA dyes, whereby bacteria strains exist that have a very high intrinsic propensity for Syto 13 staining. These bacteria absorb residual Syto 13 in the phage preparations, potentially leading to false positives or failure in the detection of phage binding if the background unspecific fluorescence of the host bacteria is already very high. For *L. lactis* F7/2, the inclusion of an additional incubation step after the bacteria were diluted into PAM allowed unspecifically bound dye, as well as bacteriophages, to dissociate from the bacteria. With this “destaining” step, it was possible to distinguish between binding by its probate phage P008 and unspecific staining by unrelated phage JG004 (Supplementary Figure S3). Thus, although the method for phage-tagging and live/dead analysis for the rapid monitoring of bacteriophage infection appears to be universally applicable, some bacteria- and phage-specific modifications may be necessary.

To summarize, in order to be certain that a positive fluorescence is indicative of an infection, this method thus currently requires rigorous controls for unspecific staining. We have outlined a few methods for such controls in this study in the form of bacteriophage infection inhibitors (e.g., sodium azide), use of related non-host bacteria strains and use of unrelated tagged phages. Due to the diversity of bacteria and bacteriophages, it might be necessary to combine and optimize various approaches to quantify bacteriophage binding and infection. There might be other more suitable DNA dyes that give lower backgrounds or are better depleted. Additionally, more refined bacteriophage purification techniques, e.g., using density gradient ultracentrifugation or stronger detergents, should still be investigated to determine if these are able to mitigate unspecific staining. Any additional processing steps, however, will have to be balanced with workability, ease of use, and preservation of bacteriophage activity.

This study also outlines a simple flow cytometric live/dead assay to quickly determine phage susceptibility. The live count obtained flow cytometrically after 10 min incubation with the live/dead stain is comparable to the plate count obtained after overnight incubation. The plate counts for *A. johnsonii* L2-215 and *E. coli* ATCC 25922, however, tend to be somewhat higher than the flow cytometric live counts, possibly due to inevitable delays associated with plating serial dilutions of bacteria during which further cell division could likely have occurred. The decrease in live count in the host *P. fluorescens* DSM 50090 in the presence of its cognate bacteriophage PMBT14, however, correlated well to a decrease in plate count.

With the rising tide of antimicrobial resistance (AMR) bacteria, there is an increasing urgency in exploring new lines of treatment for AMR bacteria. A fast screening for suitable phages for use in biocontrol or biotherapeutics may be crucial to the success of bacteriophage treatment. Foodstuffs with an acute contamination and short shelf life, or patients suffering from sepsis, generally would benefit from a fast bacteriophage screening and match. Using current methods, i.e., cultivation methods such as spot and plaque assays, phagograms (the phage susceptibility of a target bacteria) are typically obtained after the bacteria had the time to grow to confluence, typically within 18 h (Moelling et al., 2018). This is in addition to the time needed to isolate the bacteria, as well as to amplify and purify the bacteriophage preparation for end use. It is therefore imperative that fast alternative methods are developed to screen bacteriophages. The bacteriophage tagging and live–dead staining methods outlined in this paper are a step in such a direction.

The “filtering method” to remove extraneous dyes established in this study should theoretically also lend itself to SYBR Green I and other membrane permeant dyes. As a membrane filtration step is quite often incorporated into the purification process of many viruses, it remains to be determined how far this method can also be applied to other bacteriophages, as well as to enveloped and non-enveloped viruses. As flow cytometric methods are amenable to high throughput analyses, detailed information about phage binding and lysis can become much faster and easier to obtain, making characterization of newly discovered bacteriophages and phage libraries more efficient. This could in future contribute to identifying determinants of bacteriophage therapy success (Bull and Gill, 2014).

## Data Availability Statement

The original contributions presented in the study are included in the article/Supplementary Material, further inquiries can be directed to the corresponding author/s.

## Author Contributions

HL conceived the preliminary idea for the study. HL, SS, CB, JK, GF, CF, and NW were involved in the conception and design of the study. HL performed the experiments, acquired, analyzed, and interpreted the data. HL, SS, CB, JK, GF, CF, and NW were involved in the drafting and revision of the manuscript. All authors contributed to the article and approved the submitted version.

## Conflict of Interest

The authors declare that the research was conducted in the absence of any commercial or financial relationships that could be construed as a potential conflict of interest.

## References

[B1] Abuladze T. Li M. Menetrez M. Y. Dean T. Senecal A. Sulakvelidze A. (2008). Bacteriophages reduce experimental contamination of hard surfaces, tomato, spinach, broccoli, and ground beef by *Escherichia coli* O157:H7. *Appl. Environ. Microbiol.* 74 6230–6238. 10.1128/AEM.01465-08 18723643 PMC2570303

[B2] Bruttin A. Brüssow H. (2005). Human volunteers receiving *Escherichia coli* phage T4 orally: a safety test of phage therapy. *Antimicrob. Agents Chemother.* 49 2874–2878. 10.1128/AAC.49.7.2874-2878.2005 15980363 PMC1168693

[B3] Bull J. J. Gill J. J. (2014). The habits of highly effective phages: population dynamics as a framework for identifying therapeutic phages. *Front. Microbiol.* 5:618. 10.3389/fmicb.2014.00618 25477869 PMC4235362

[B4] Chan B. K. Abedon S. T. Loc-Carrillo C. (2013). Phage cocktails and the future of phage therapy. *Fut. Microbiol.* 8 769–783. 10.2217/fmb.13.47 23701332

[B5] Dang V. T. Sullivan M. B. (2014). Emerging methods to study bacteriophage infection at the single-cell level. *Front. Microbiol.* 5:724. 10.3389/fmicb.2014.00724 25566233 PMC4274963

[B6] Dedrick R. M. Guerrero-Bustamante C. A. Garlena R. A. Russell D. A. Ford K. Harris K. (2019). Engineered bacteriophages for treatment of a patient with a disseminated drug-resistant Mycobacterium abscessus. *Nat. Med.* 25 730–733. 10.1038/s41591-019-0437-z 31068712 PMC6557439

[B7] Deng L. Gregory A. Yilmaz S. Poulos B. T. Hugenholtz P. Sullivan M. B. (2012). Contrasting life strategies of viruses that infect photo- and heterotrophic bacteria, as revealed by viral tagging. *mBio* 3:e00373-12. 10.1128/mBio.00373-12 23111870 PMC3487772

[B8] Dlusskaya E. A. Atrazhev A. M. Ashbolt N. J. (2019). Colloid chemistry pitfall for flow cytometric enumeration of viruses in water. *Water Res. X* 2:100025. 10.1016/j.wroa.2019.100025 31194069 PMC6549941

[B9] Gindin M. Febvre H. P. Rao S. Wallace T. C. Weir T. L. (2019). Bacteriophage for Gastrointestinal Health (PHAGE) Study: evaluating the safety and tolerability of supplemental bacteriophage consumption. *J. Am. Coll. Nutr.* 38 68–75. 10.1080/07315724.2018.1483783 30157383

[B10] Goodridge L. Chen J. Griffiths M. (1999). Development and Characterization of a Fluorescent-Bacteriophage Assay for Detection of *Escherichia coli* O157:H7. *Appl. Environ. Microbiol.* 65 1397–1404. 10.1128/aem.65.4.1397-1404.1999 10103228 PMC91198

[B11] Gordillo Altamirano F. L. Barr J. J. (2019). Phage therapy in the postantibiotic era. *Clin. Microbiol. Rev.* 32:25. 10.1128/CMR.00066-18 30651225 PMC6431132

[B12] Grant A. ’Q. Parveen S. Schwarz J. Hashem F. Vimini B. (2017). Reduction of *Salmonella* in ground chicken using a bacteriophage. *Poult. Sci.* 96 2845–2852. 10.3382/ps/pex062 28371846

[B13] Havelaar A. H. Kirk M. D. Torgerson P. R. Gibb H. J. Hald T. Lake R. J. (2015). World health organization global estimates and regional comparisons of the burden of foodborne disease in 2010. *PLoS Med.* 12:e1001923. 10.1371/journal.pmed.1001923 26633896 PMC4668832

[B14] IACG (2019). No Time To Wait: Securing The Future From Drug-Resistant Infections. IAGG Available online at: https://www.who.int/antimicrobial-resistance/interagency-coordination-group/final-report/en/

[B15] Janczuk M. Richter Ł Hoser G. Kawiak J. Łoś M. Niedziółka-Jönsson J. (2017). Bacteriophage-Based Bioconjugates as a Flow Cytometry Probe for Fast Bacteria Detection. *Bioconjug. Chem.* 28 419–425. 10.1021/acs.bioconjchem.6b00596 27990800

[B16] Koberg S. Gieschler S. Brinks E. Wenning M. Neve H. Franz C. M. A. P. (2018). Genome sequence of the novel virulent bacteriophage PMBT14 with lytic activity against *Pseudomonas* fluorescens DSM 50090R. *Arch. Virol.* 163 2575–2577. 10.1007/s00705-018-3882-y 29786121

[B17] Moelling K. Broecker F. Willy C. (2018). A wake-up call: we need phage therapy now. *Viruses* 10:688. 10.3390/v10120688 30563034 PMC6316858

[B18] Mosier-Boss P. A. Lieberman S. H. Andrews J. M. Rohwer F. L. Wegley L. E. Breitbart M. (2003). Use of fluorescently labeled phage in the detection and identification of bacterial species. *Appl. Spectrosc.* 57 1138–1144. 10.1366/00037020360696008 14611044

[B19] Moye Z. D. Woolston J. Sulakvelidze A. (2018). Bacteriophage Applications for Food Production and Processing. *Viruses* 10:205. 10.3390/v10040205 29671810 PMC5923499

[B20] Müller-Merbach M. Neve H. Hinrichs J. (2005). Kinetics of the thermal inactivation of the Lactococcus lactis bacteriophage P008. *J. Dairy Res.* 72 281–286. 10.1017/S0022029905000725 16174358

[B21] Ostergaard Breum S. Neve H. Heller K. J. Vogensen F. K. (2007). Temperate phages TP901-1 and phiLC3, belonging to the P335 species, apparently use different pathways for DNA injection in Lactococcus lactis subsp. *cremoris* 3107. *FEMS Microbiol. Lett.* 276 156–164. 10.1111/j.1574-6968.2007.00928.x 17956421

[B22] Perera M. N. Abuladze T. Li M. Woolston J. Sulakvelidze A. (2015). Bacteriophage cocktail significantly reduces or eliminates Listeria monocytogenes contamination on lettuce, apples, cheese, smoked salmon and frozen foods. *Food Microbiol.* 52 42–48. 10.1016/j.fm.2015.06.006 26338115

[B23] Sambrook J. Russell D. W. (2001). *Molecular Cloning: A Laboratory Manual.* Cold Spring Harbor, NY: Cold Spring Harbor Laboratory Press.

[B24] Sarker S. A. Berger B. Deng Y. Kieser S. Foata F. Moine D. (2017). Oral application of *Escherichia coli* bacteriophage: safety tests in healthy and diarrheal children from Bangladesh. *Environ. Microbiol.* 19 237–250. 10.1111/1462-2920.13574 27750388

[B25] Sarker S. A. McCallin S. Barretto C. Berger B. Pittet A.-C. Sultana S. (2012). Oral T4-like phage cocktail application to healthy adult volunteers from Bangladesh. *Virology* 434 222–232. 10.1016/j.virol.2012.09.002 23102968

[B26] Schooley R. T. Biswas B. Gill J. J. Hernandez-Morales A. Lancaster J. Lessor L. (2017). Development and use of personalized bacteriophage-based therapeutic cocktails to treat a patient with a disseminated resistant *acinetobacter baumannii* infection. *Antimicrob. Agents Chemother.* 61:e000954-17. 10.1128/AAC.00954-17 28807909 PMC5610518

[B27] Soni K. A. Nannapaneni R. Hagens S. (2010). Reduction of Listeria monocytogenes on the surface of fresh channel catfish fillets by bacteriophage Listex P100. *Foodborne Pathog. Dis.* 7 427–434. 10.1089/fpd.2009.0432 19958102

[B28] Stocks S. M. (2004). Mechanism and use of the commercially available viability stain. *BacLight. Cytometry A* 61 189–195. 10.1002/cyto.a.20069 15382024

[B29] Sukumaran A. T. Nannapaneni R. Kiess A. Sharma C. S. (2015). Reduction of *Salmonella* on chicken meat and chicken skin by combined or sequential application of lytic bacteriophage with chemical antimicrobials. *Int. J. Food Microbiol.* 207 8–15. 10.1016/j.ijfoodmicro.2015.04.025 25950852

[B30] Wright A. Hawkins C. H. Anggård E. E. Harper D. R. (2009). A controlled clinical trial of a therapeutic bacteriophage preparation in chronic otitis due to antibiotic-resistant *Pseudomonas aeruginosa*; a preliminary report of efficacy. *Clin. Otolaryngol.* 34 349–357. 10.1111/j.1749-4486.2009.01973.x 19673983

[B31] Wu L. Song Y. Luan T. Ma L. Su L. Wang S. (2016). Specific detection of live *Escherichia coli* O157:H7 using tetracysteine-tagged PP01 bacteriophage. *Biosens Bioelectr.* 86 102–108. 10.1016/j.bios.2016.06.041 27341136

