# Supplementary material for: Fast and Easy Phage-Tagging and Live/Dead Analysis for the Rapid Monitoring of Bacteriophage Infection
Source: Front Microbiol. 2020 Dec 18;11:602444. doi: 10.3389/fmicb.2020.602444 (PMC7775415; doi:10.3389/fmicb.2020.602444)

Supplementary Material

Supplementary Figure Legend

Supplementary Figure 1. Fluorescence measurement of PMBT14+Syto 13 or Syto 13 in the presence of 0 (Blank), 5 ng, 25 ng or 50 ng of DNA. PMBT14+Syto 13 samples were analyzed before filtration (unfiltered) or after filtration through a 0.45 µm PES membrane (0.45 µm filtered). ΔEm510 is defined as the fluroescence difference between the mean intrinsic fluorescence of the sample, i.e. without any additional DNA, and the mean fluorescence after addition of 50 ng extraneous DNA. The 0.45 µm filtered samples were additionally filtered through a 0.2 µm PES membrane (0.45 µm + 0.2 µm filtered) or lysed at 95°C for 5 min (0.45 µm filtered, lysed). Syto 13 in SMG-T buffer was analysed before and after 0.45 µm filtration. Bars are the mean fluorescence with standard error of measurement and are derived from triplicate measurements over 2 experiments.

Supplementary Figure 2. Time lapse CLSM of PMBT14-Syto 13 binding to and infection of its host, *P. fluorescens* L1-81, with the orange arrows pointing to 2 phages that have given up their DNA in the course of 30 min as their fluorescence visibly decreases (row 1). The infection is inhibited in the presence of 1% sodium azide (row 2). In *P. fluorescens* L1-82 (row 3), binding occurred with no infection, whereas in *E. coli* L1-21, neither binding nor infection occurred (row 4). Orange arrows serve to point to bacteriophages bound to bacteria.

Supplementary Figure 3. Staining of *P. aeruginosa* PAO 1 DSM 22644 with its cognate phage JG004 tagged with Syto 13. Phage P008 was used as unspecific background control, *P. fluorescens* DSM 50090 was used as non-host control to control for similar amount of tagged bacteriophage preparation. In order to gate out dead bacteria that can bind unspecifically to Syto 13, PI was used in the acquisition buffer. Bacteria was first gated by their FSC and SSC characteristics, and then dead cells that were PI positive were excluded from analysis. Geometric mean fluorescence intensities are given next to each peak.

Supplementary Figure 4. Staining of *L. lactis* F7/2 with its cognate phage P008 tagged with Syto 13. Phage JG004 was used as unspecific background control. *P. fluorescens* DSM 50090 was used as non-host control to control for similar amount of tagged bacteriophage preparation. Bacteria were gated by their FSC and SSC characteristics. Geometric mean fluorescence intensities are given next to each peak.

Supplementary Videos. 3D projection of Z-stack acquired via CLSM after 35 min incubation of PMBT14-Syto 13 with bacteria (Figure 2B).

Supplementary Figure 1

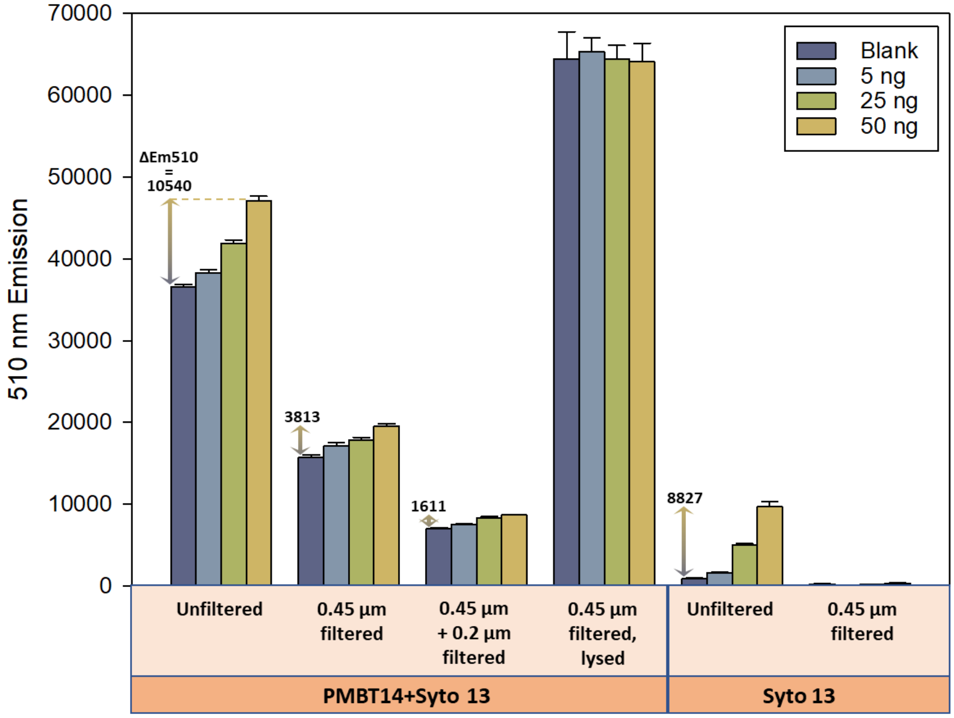

Supplementary Figure 2

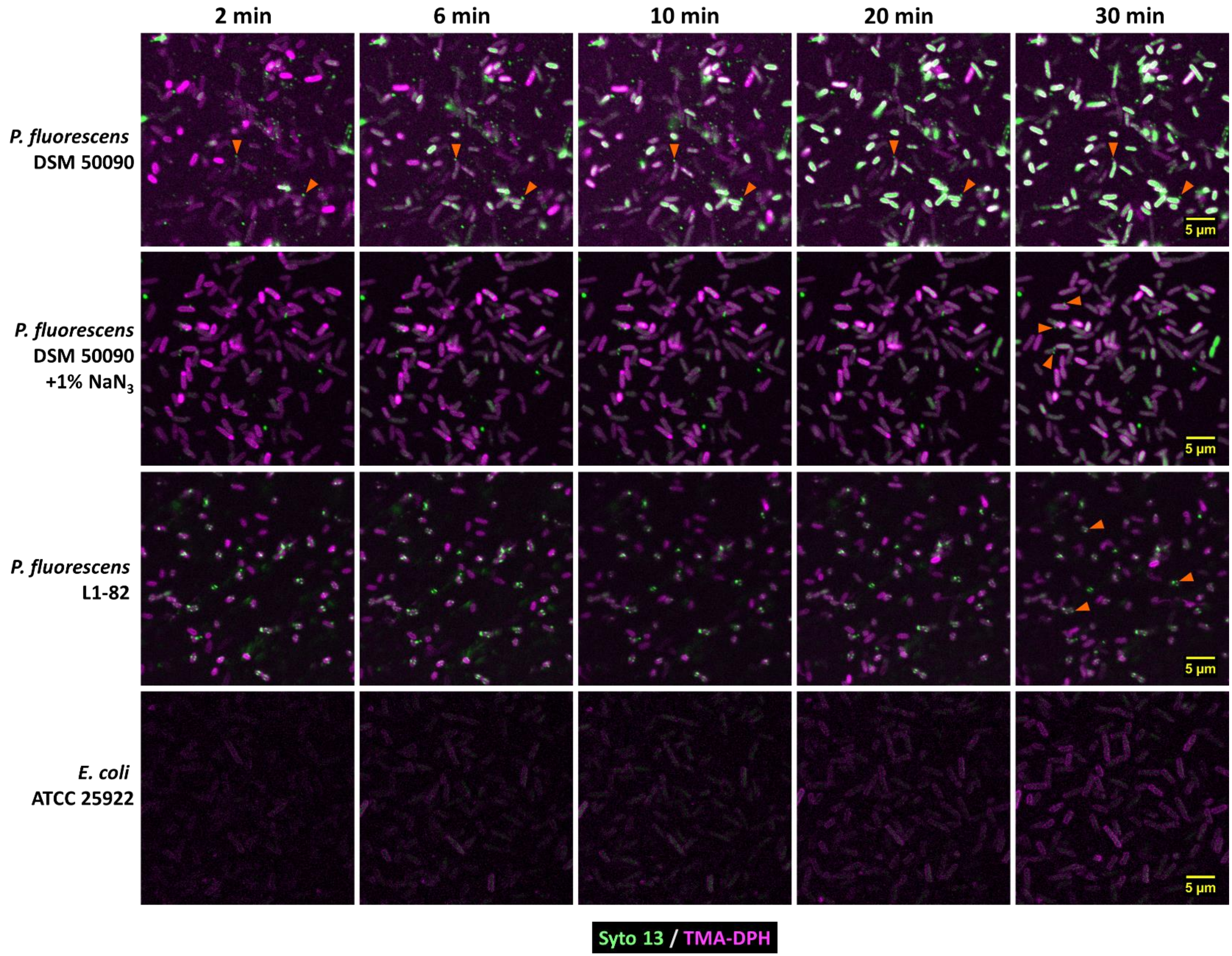

Supplementary Figure 3

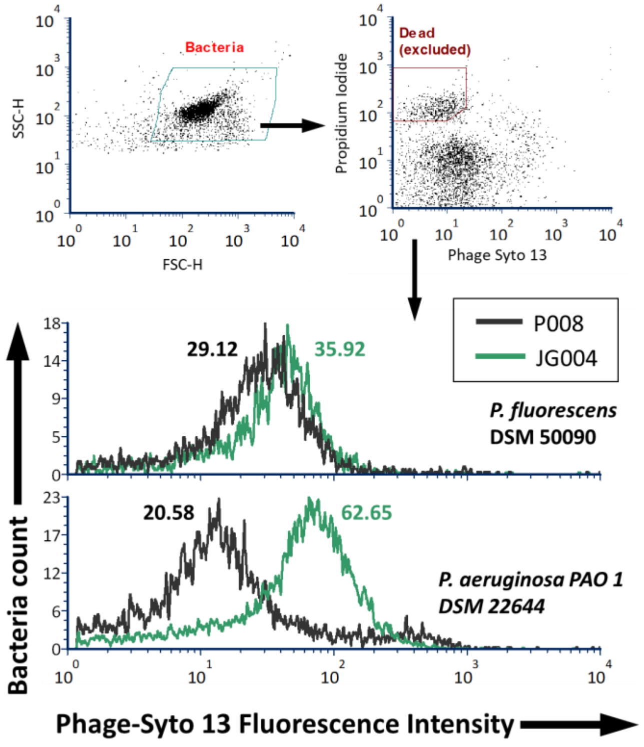

Supplementary Figure 4

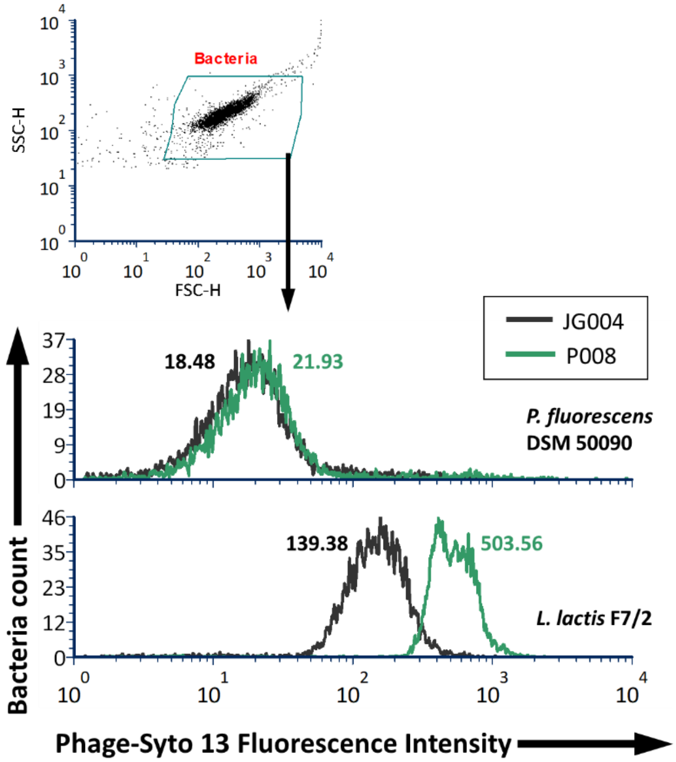

Supplement: Supplementary file 1 [file Data_Sheet_1.pdf]
